# *CDH23*-Associated Usher Syndrome: Clinical Features, Retinal Imaging, and Natural History

**DOI:** 10.1167/iovs.65.8.27

**Published:** 2024-07-17

**Authors:** Thales A. C. de Guimaraes, Anthony G. Robson, Isabela M. C. de Guimaraes, Yannik Laich, Nancy Aychoua, Genevieve Wright, Angelos Kalitzeos, Omar A. Mahroo, Andrew R. Webster, Michel Michaelides

**Affiliations:** 1 UCL Institute of Ophthalmology, University College London, London, United Kingdom; 2 Moorfields Eye Hospital NHS Foundation Trust, London, United Kingdom; 3 Universidade Sao Leopoldo Mandic, Campinas, Sao Paulo, Brazil; 4 Eye Center, Faculty of Medicine, University Freiburg, Germany

**Keywords:** retinitis pigmentosa (RP), rod-cone dystrophy, inherited retinal dystrophy, *CDH23*, Usher syndrome (USH), natural history

## Abstract

**Purpose:**

The purpose of this study was to analyze the clinical spectrum and natural history of *CDH23*-associated Usher syndrome type ID (USH1D).

**Methods:**

Molecularly-confirmed individuals had data extracted from medical records. Retinal imaging was extracted from an in-house database. The main outcome measurements were retinal imaging and electroretinography (ERG) and clinical findings, including age of onset, symptoms, best-corrected visual acuity (BCVA), outer nuclear layer (ONL) thickness, ellipsoid zone width (EZW), and hyperautofluorescent ring area.

**Results:**

Thirty-one patients were identified, harboring 40 variants in *CDH23* (10 being novel). The mean (range, ±SD) age of symptom onset was 10.1 years (range = 1–18, SD = ±4.1). The most common visual symptoms at presentation were nyctalopia (93.5%) and peripheral vision difficulties (61.3%). The mean BCVA at baseline was 0.25 ± 0.22 in the right eyes and 0.35 ± 0.58 LogMAR in the left eyes. The mean annual loss rate in BCVA was 0.018 LogMAR/year over a mean follow-up of 9.5 years. Individuals harboring the c.5237G>A p.(Arg1746Gln) allele had retinitis pigmentosa (RP) sparing the superior retina. Seventy-seven percent of patients had hyperautofluorescent rings in fundus autofluorescence. Full-field and pattern ERGs indicated moderate-severe rod-cone or photoreceptor dysfunction with relative sparing of macular function in most patients tested. Optical coherence tomography (OCT) revealed intraretinal cysts in the transfoveal B-scan of 13 individuals (43.3%). The rate of EZW and ONL thickness loss was mild and suggestive of a wide window of macular preservation.

**Conclusions:**

Despite the early onset of symptoms, USH1D has a slowly progressive phenotype. There is high interocular symmetry across all parameters, making it an attractive target for novel therapies.

Usher syndrome (USH) refers to an heterogenous group of disorders inherited in a recessive fashion, that is the most common cause of combined hearing and vision loss, with or without vestibular dysfunction, with a prevalence ranging from 1 to 4 of 25,000.^1^^,^^2^ It has been historically subdivided into three groups (USH1, USH2, and USH3) according to the severity of hearing loss, onset of retinitis pigmentosa (RP) and presence/absence of vestibular dysfunction. A fourth and atypical subgroup (USH4) was further identified and associated with variants in *ARSG* (OMIM *618144), which has also recently been expanded to *CEP78* (OMIM *617110), *CEP250* (OMIM *609689), and *ABHD12* (OMIM *613599).^3^^,^^4^

USH1D is caused by variants in *CDH23* (OMIM #601067), which was mapped to chromosome 10q in 1996 by Wayne and collaborators.^5^ Variants have also been reported to cause digenic USH1 if associated with pathogenic heterozygous variants in *PCDH15* (OMIM #605514). USH1D accounts for 19% to 35%^4^^,^^5^ of USH1 cases, and, as reported in a systematic metanalysis published in 2019, pathogenic variants in *CDH23* were present in 6% (39/684) of patients with combined visual and hearing impairment due to USH, making it the third most common cause of dual sensory impairment (DSI) in affected individuals.^6^^–^^8^ This gene is a member of the cadherin superfamily which is expressed in both the outer and inner hair cells in the cochlea.^9^ Affected individuals have profound congenital hearing impairment, RP within the first decade of life, and frequent vestibular dysfunction.

USH1D has been well characterized from a hearing loss perspective and variants in *CDH23* have been identified as a major cause of non-syndromic sensorineural hearing loss.^10^ However, there is scarce evidence in the literature of (i) the retinal phenotype of *CDH23*-associated USH1, both functionally and structurally, as well as its (ii) natural history, nor well-defined, robust (iii) retinal genotype-phenotype correlations. This is crucial data to improve counselling of affected patients and families and could also be used as a framework for developing novel treatments in the future. The aim of this study is to report the clinical features and the structural changes in the retina cross-sectionally and longitudinally.

## Materials and Methods

This retrospective cohort study conformed to the tenets of the Declaration of Helsinki and was approved by the Moorfields Eye Hospital ethics committee. All patients included in this database had provided informed consent previously.

### Patient Identification

All patients previously identified with molecularly confirmed USH1D in a tertiary referral center (Moorfields Eye Hospital, London, UK) were reviewed. The patients were identified using an in-house database (OpenEyes, London, UK). Information was then extracted from electronic healthcare records and physical medical notes.

### Genetic Testing

As part of routine clinical diagnostics, a combination of Sanger direct sequencing and/or next-generation sequencing, sequencing panels of retinal dystrophy genes, whole-exome sequencing (WES), and whole-genome sequencing (WGS) was used to identify variants in *CDH23*.

### Clinical Data

All patients were seen by experienced inherited retinal disease specialists. Available clinical notes were reviewed, including medical history, best-corrected visual acuity (BCVA), refraction, fundoscopy, and slit-lamp biomicroscopy findings. Age of onset was defined as the age in which the vision-related symptoms started. BCVA analysis was performed cross-sectionally and longitudinally and will be expressed as LogMAR using the reference provided by Day et al. (2015).^11^ Spherical equivalent was calculated for refractive errors.

### Retinal Imaging

Optical coherence tomography (OCT) and fundus autofluorescence (FAF) were obtained using the Spectralis SD-OCT (Heidelberg Engineering Inc., Heidelberg, Germany), and ultra-widefield pseudocolor imaging and FAF were acquired with the Optos imaging system (Optos plc, Dunfermline, UK). There was no standardization in the acquisition of SD-OCT images, with the nominal extent being a mix of 20- and 30-degree B-scans. High resolution mode was used whenever possible.

Both OCT and FAF were scrutinized quantitatively by using the digital calipers provided in the Heyex version 2.0 (Heidelberg Eye Explorer; Heidelberg Engineering). The imaging was graded by an experienced physician (author T.A.C.G.) using maximum magnification and a 1 µm:1 µm display to quantify features in the transfoveal horizontal B-scan. Whenever present, the foveal reflex was used as an anatomic landmark. The extent of the preserved ellipsoid zone width (EZW) was measured after marking the temporal and nasal boundaries of the EZ for accuracy. The outer nuclear layer (ONL) thickness was also measured as the distance between the outer plexiform layer and external limiting membrane, except for cases with intraretinal cystic spaces (IRCS) in the transfoveal scan due to difficulties in clearly delimitating the ONL. Similarly, the area within the hyperautofluorescent ring was quantified manually in the FAF images by using the area tool and tracing the total area from the external border of the hyperautofluorescent ring, as described in a recent study.^12^ The authors used 55 × 55 degree images given the large area of some rings which would otherwise not be measurable in the 30 × 30 degree images. In cases where a double ring was present, only the area of the outer most ring was measured. Follow-up mode was used to allow longitudinal analysis of the same location. These methods are described in detail in two previous studies.^13^^,^^14^

### Visual Electrophysiology

Full-field and pattern electroretinogram (PERG; ERG) testing was performed in nine patients, incorporating the standards of the International Society for Clinical Electrophysiology of Vision (ISCEV), using gold foil corneal recording electrodes.^15^^,^^16^ Full-field ERGs were used to assess generalized rod and cone system function and pattern ERG P50 was used to quantify macular function.^17^ The ERG data were compared with a reference range from a group of healthy subjects (age range = 10–79 years).^18^^,^^19^ The amplitudes of the main ISCEV Standard ERG components were plotted as a percentage of the age-matched lower limit (5th percentile) of the reference range or as a peak time difference from the age-matched upper limit (95th percentile). In addition, four children (ages 3, 4, 6, and 9 years) were tested according to a shortened protocol (Holder and Robson 2006) using lower eyelid skin electrodes with Ganzfeld (*n* = 3) or non-Ganzfeld flashes (*n* = 1).^20^ Three of the four children underwent PERG testing with lower eyelid skin electrodes and one using gold foil electrodes.

### Statistical Methods

Statistical analysis was performed with GraphPad Prism 9 (GraphPad Software; San Diego, CA, USA). Parametric and nonparametric tests were used, as well as correlation parameters (either Pearson or Spearman). Significance of all statistical tests was set at *P* < 0.05 and D'Agostino-Pearson test (omnibus K2) was used to determine normality for all variables. All variables were analyzed for both eyes, but in the case of high interocular symmetry, only the right eye was included in subsequent analyses for clarity.

## Results

In total, 31 patients of 29 families with USH1D were identified. The cohort comprised of 19 female patients (61.3%) and 12 male patients (38.7%). Twenty-nine patients had longitudinal data available. The Table provides a summary of the main clinical and molecular findings of the cohort.

**Table.** Summary of all Alleles (c.DNA Changes) and Phenotypes

ID	Allele 1	Allele 2	Phenotype	Age of Onset (Years)	Presenting Visual Symptoms
MEH001	c.4552delC	c.4552delC	RP	8	Nyctalopia and peripheral vision issues
MEH002	c.8480_8481del*	c.8480_8481del*	RP	Unknown	Nyctalopia and peripheral vision issues
MEH003	c.8588-8611dup*	c.8588-8611dup*	RP	8	Peripheral vision issues
MEH004	Exon 4-6 del*	Exon 4_6 del*	RP	9	Nyctalopia and peripheral vision issues
MEH005	c.5237G>A	c.9278+2T>G	Sectoral RP	17	Mild peripheral vision issues
MEH006	c.3954C>A	Exon 17_30 del*	RP	6	Nyctalopia and peripheral vision issues
MEH007	c.6393delC	c.6254-3_6254delinsT	RP	7	Peripheral vision issues
MEH008	c.6900C>G	c.9077+1G>T	RP	14	Nyctalopia
MEH009	c.5237G>A	c.7823G>A	Sectoral RP	Asymptomatic	Asymptomatic
MEH010	c.1411G>A	c.4759_4766del8	RP	6	Nyctalopia
MEH011	c.8308+1G>A	c.336+1del	RP	10	Nyctalopia
MEH012	c.7482+2T>C	c.5916_5917delTC	RP	unknown	Nyctalopia
MEH013	Exon 67_69 del*	Exon 67_69 del*	RP	Unknown	Nyctalopia and peripheral vision issues
MEH014	c.6319C>T	c.7305dup*	RP	8	Unknown
MEH015	c.8722G>A	c.3337G>C	RP	Unknown	Nyctalopia and peripheral vision issues
MEH016	c.2398-1G>T	c.7908C>G	RP	8	Nyctalopia
MEH017	c.4246C>T*	c.4246C>T*	RP	9	Nyctalopia and peripheral vision issues
MEH018	c.1336delG*	c.9122T>C	RP	12	Nyctalopia and peripheral vision issues
MEH019	c.5368+1G>T	c.5237G>A	RP	Unknown	Too young for symptoms
MEH020	Exon 67_68 del	Exon 67_68 del	RP	13	Nyctalopia and peripheral vision issues
MEH021	c.7823G>A	c.9122T>C	RP	12	Nyctalopia and peripheral vision issues
MEH022	c.7908C>G	c.193delC	RP	10	Nyctalopia
MEH023	c.6319C>T	c.6319C>T	RP	Unknown	Nyctalopia
MEH024	c.1986+3A>T*	c.1986+3A>T*	RP	1	Nyctalopia and peripheral vision issues
MEH025	c.1986+3A>T*	c.1986+3A>T*	RP	unknown	Nyctalopia
MEH026	c.9122T>C	c.2177-2A>G	RP	unknown	Nyctalopia
MEH027	c.4759_4766del	Not found	RP	18	Nyctalopia and peripheral vision issues
MEH028	c.1369C>T	c.5237G>A	Sectoral RP	16	Peripheral vision issues
MEH029	c.1411G>A	c.4759_4766del8	RP	16	Nyctalopia and peripheral vision issues
MEH030	c.6050-9G>A	c.6050-9G>A	RP	8	Nyctalopia and peripheral vision issues
MEH031	c.9319+1G>T	Exon 35_38 del*	RP	14	Nyctalopia and peripheral vision issues

Ten variants are novel (asterisks).

RP, retinitis pigmentosa.

### Molecular Variants

Forty disease-causing variants were identified, of which 10 were missense and another 10 were splice-site variants (25% each), 6 were out-of-frame intragenic deletions (15%), 5 were nonsense variants (12.5%), 4 were deletions of entire exons (10%), 2 were intragenic duplications (5%), 1 was intronic, another was an in-frame intragenic deletion/single amino acid deletion, and there was also a del-ins that generated a complex protein rearrangement with a premature termination (2.5% each). To the best of our knowledge, 10 variants reported herein were novel (see the Table).

The most frequent variants were c.5237G>A p.(Arg1746Gln) and c.9122T>C p.(Leu3041Pro), which were present in 4 and 3 alleles of unrelated families, respectively. Only one variant was found in patient MEH027 (both in a large panel of retinal dystrophy genes and subsequent, WGS, but he was included herein given the secure clinical diagnosis of USH type I.

### Disease Onset and Symptoms

Twenty-one patients had the approximate age of onset of vision-related symptoms recorded. The mean (range; ±SD) age of symptom onset was 10.2 years (range = 1–18, SD = ±4.1).

The most common presenting visual symptoms in our cohort at onset were nyctalopia (93.5%) and difficulties with peripheral vision (61.3%). Four patients developed photophobia during the course of the disease, 3 other patients had low amplitude, high frequency nystagmus noticed around the age of 1 year, and another patient (MEH005) remained completely asymptomatic until his most recent follow-up at 21 years of age. Another patient (MEH004) had exotropia noticed at 5 years of age and corrected with surgery at 10 years. All patients had sensorineural hearing loss at birth, but interestingly, one patient (MEH009) had only mild hearing difficulties and did not require cochlear implants.

### Clinical Features

**Figure 1.**
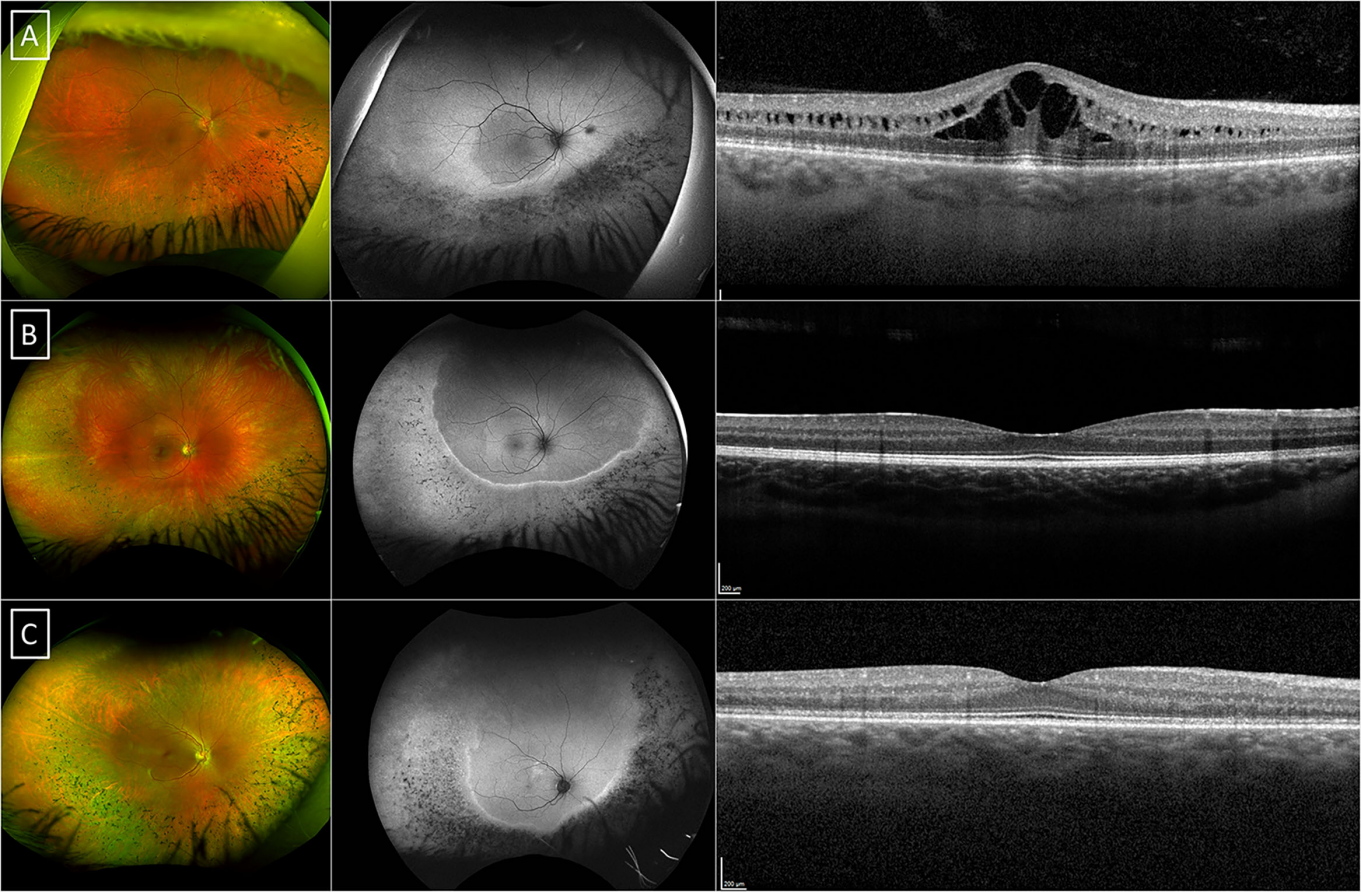
**Multimodal imaging of the patients harbo** **ring the c.5237G>A p.(Arg1746Gln) variant.** Pseudocolor wide-field fundus photography, fundus autofluorescence (FAF) and optical coherence tomography (OCT) of the three patients with sectoral-like retinitis pigmentosa, MEH005 (**A**), MEH009 (**B**), and MEH028 (**C**). There is a sharp hyper-autofluorescent ring providing demarcation of the affected areas of the retina, sparing the superior quadrant in three patients.

On ocular examination, posterior subcapsular cataracts were common, which were first noticed in 11 patients (38.7%) at a mean age (range; ±SD) of 32.2 years (range = 25–48, SD = ±8.3). In most patients, the fundus had typical findings of RP, with bony spicules and RPE mottling in the mid-periphery, and a preserved central retina. MEH006 had poor vision in the left eye from childhood due to a chronic retinal detachment after a head trauma, and MEH022 had a left macular lamellar hole. Three patients had a phenotype (MEH005, MEH009, and MEH028) with bony spicules mainly in the inferior quadrants, with the superior retina largely spared, all harboring the c.5237G>A p.(Arg1746Gln) variant in compound heterozygosity (Fig. 1). Their phenotype is interesting and more in keeping with a preservation of the superior quadrant of the retina as opposed to the typical sectoral RP usually restricted to the inferonasal quadrant.^21^^,^^22^ A similar phenotype has been described in *EYS*-associated RP.^23^

### Refraction

Refractive data was available for all patients, but three patients were referred to us post-cataract surgery (i.e. pseudophakic). The mean age (range; ±SD) when refraction was obtained was 22.7 years (range = 3–49, SD = ±14.7). The mean spherical equivalent was −0.85 diopters (D; range = −4.00 to +7.5, SD = ±2.6) in the right eyes and −0.78 D (range = −3.75 to +8.5, SD = ±2.85) in the left eyes. The most frequent type of refractive error was myopic astigmatism, which was seen in 10 individuals (35.7%).

### Visual Acuity

BCVA was assessed cross-sectionally at baseline (*n* = 29) and longitudinally (*n* = 28). Given the aforementioned history of other diagnosis in the left eye, MEH006 and MEH022 were removed completely from this analysis. None of the other patients had any other significant vision-limiting conditions.

**Figure 2.**
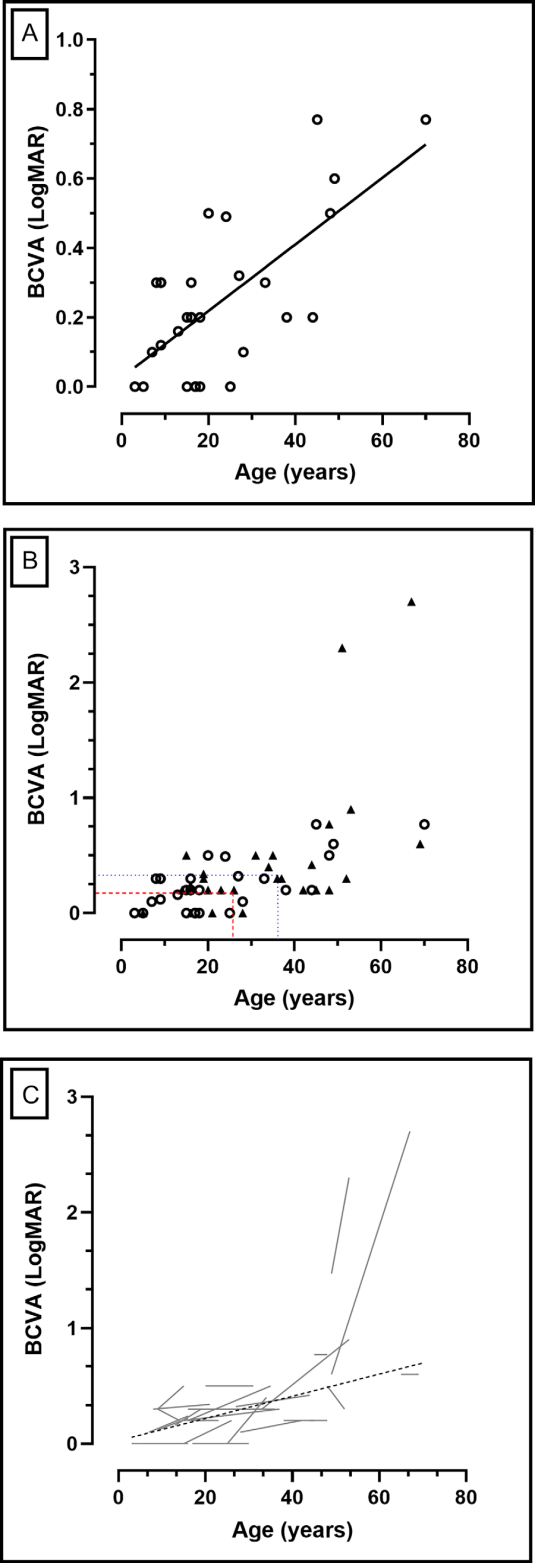
**Best corrected visual acuity (BCVA) changes over time.** (**A**) Simple linear regression of BCVA at the baseline visit against age. There is a significant reduction of vision (*P* < 0.0001) with age. (**B**) Baseline and longitudinal BCVA. The *red dashed lines* show the mean age and BCVA at baseline –23.7 years and 0.28 LogMAR, respectively – whereas the *dotted blue lines* represent the mean age and BCVA at follow-up –33.2 years and 0.46 LogMAR, respectively – after a mean follow-up of 9.5 years. The mean annual loss rate in BCVA was 0.018 LogMAR/year. (**C**) The *gray lines* are representing individual progression of the right eyes of each subject in this cohort, with the overall progression (linear regression) plotted as a *dashed black line*.

The mean BCVA (range; ±SD) at baseline was 0.25 LogMAR (range = 0–0.77, SD = ±0.22) in the right eyes and 0.35 LogMAR (range = −0.1–3, SD = ±0.58) at a mean age of 22.8 years (range = 3–70, SD = ±15.9). There was a significant interocular correlation of BCVA at the baseline visit (*r* = 0.94, *P* < 0.0001; Spearman correlation). Given the high correlation, for clarity, only the values for right eyes are described subsequently.^24^ A simple linear regression was then fitted (for visual acuity against age) and revealed a slope which was significantly non-zero (*F* = 24.13, *P* < 0.0001; Fig. 2A).

Longitudinal data were then analyzed in the right eyes. The mean age (range; ±SD) at the baseline and follow-up visit was 23.7 (range = 3–65, SD = ±16.4) and 33.2 years (range = 5–69, SD = ±16.7). After a mean follow-up (±SD) of 9.5 years (SD = ±6.1), there was a change in mean BCVA from 0.28 (SD = ±0.31) to 0.46 LogMAR (SD = ±0.6), which reached statistical significance (*P* = 0.003; Wilcoxon matched-pairs signed rank test; Fig. 2B). The mean annual loss rate in BCVA was 0.018 LogMAR/year. Figure 2C shows the individual BCVA change with time for all subjects in this cohort.

### Retinal Imaging

Thirty patients had retinal imaging at baseline with a mean age (range; ±SD) of 27.4 years (range = 3–70, SD = ±18.3). Longitudinal data was available for a subset of 27 patients (mean age at last visit = 33.7 years) with a mean follow-up time of 7.2 years, which was statistically significant (*P* < 0.0001, t = 9.4, df = 26; paired *t*-test).

#### Fundus Autofluorescence

**Figure 3.**
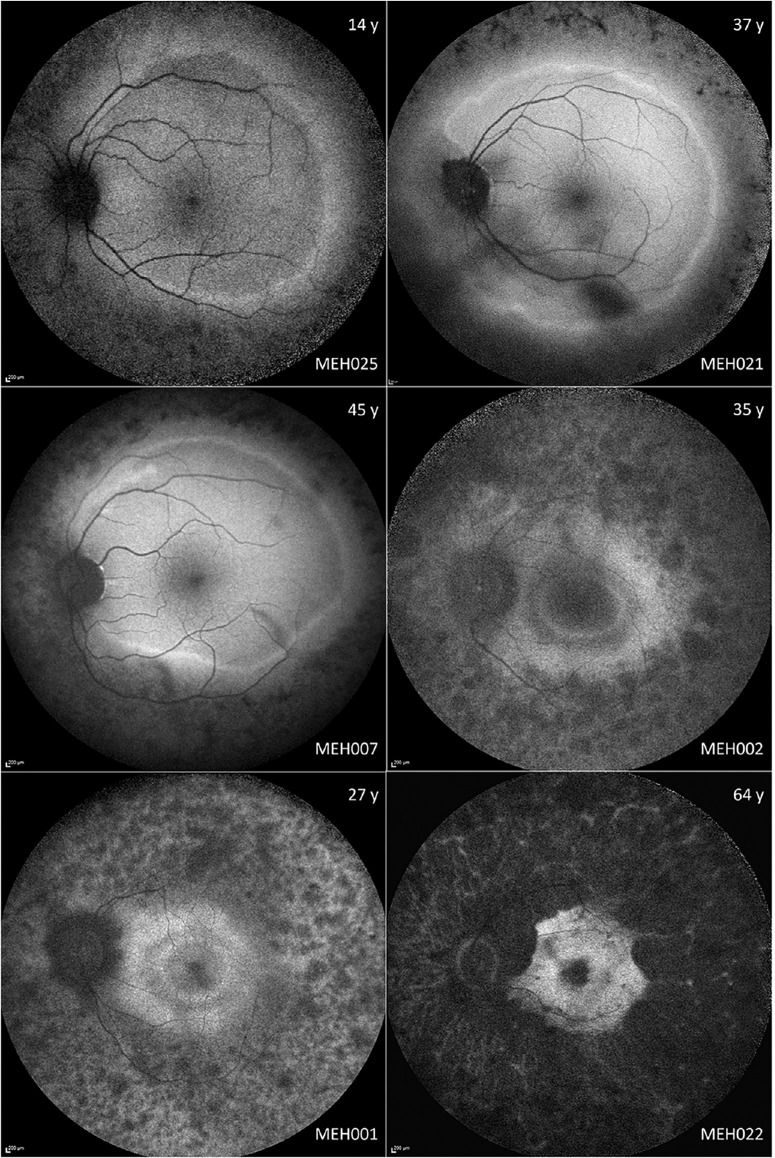
**Montage of fundus autofluorescence (FAF) imaging in different disease stages.** On the *top and bottom right corner* are the age (years) and the subject ID, respectively. The most frequent early imaging findings are hyperautofluorescent rings, which are usually located beyond the vascular arcades. With disease progression, the rings tend to gradually constrict. MEH002 and MEH001 have double hyperautofluorescent rings separated by a patch of normal autofluorescence.

There was moderate variability in the FAF appearance, ranging from normal (MEH009) to severely abnormal (MEH008; Fig. 3). However, all features described were bilateral and highly symmetric. A typical feature was the presence of a perimacular hyperautofluorescent (hyperAF) ring (*n* = 23; 77%), seven of which were beyond the vascular arcades. Most patients had a ring which was stable throughout the follow-up period, albeit two had a progressively smaller concentric ring that encroached toward the macula with disease advancement. Interestingly, five patients had double hyperAF rings that did not change. In the subjects with the sectoral-like RP phenotype, there was an incomplete hyperAF ring, deficient superiorly, in two cases. MEH005 and MEH026 had an increased central signal.

The hyperAF rings were then quantitatively assessed. Fourteen patients had bilateral FAF with fair quality at baseline, with longitudinal data being available for 11 of these. Another 8 subjects had hyperAF rings that could not be measured accurately due to image quality, whereas another subject had a ring that was too large, with borders extending beyond the limits of the 55 × 55 degrees FAF. The mean area within the hyperAF rings at baseline was 23.90 mm^2^ in the right eyes and 25.05 mm^2^ in the left eyes, which was significantly correlated (*r* = 0.99; *P* < 0.0001; Spearman correlation).

Longitudinal data was available for 11 subjects, 3 of which had hyperAF rings extending beyond the vascular arcades. The baseline area within the hyperAF ring in this subgroup was 29.73 mm^2^ in the right eyes and 29.36 mm^2^ in the left eyes. After a mean (range; ±SD) follow-up of 9.4 years (range = 4–22, SD = ±6.33), the mean area reduced to 27.62 mm^2^ and 25.11 mm^2^ in the right and left eyes, respectively, which was statistically significant (*P* < 0.0001 in both eyes). The area within the hyperAF ring reduced in all cases except in the three patients where the ring extended beyond the vascular arcades.

#### Optical Coherence Tomography

**Figure 4.**
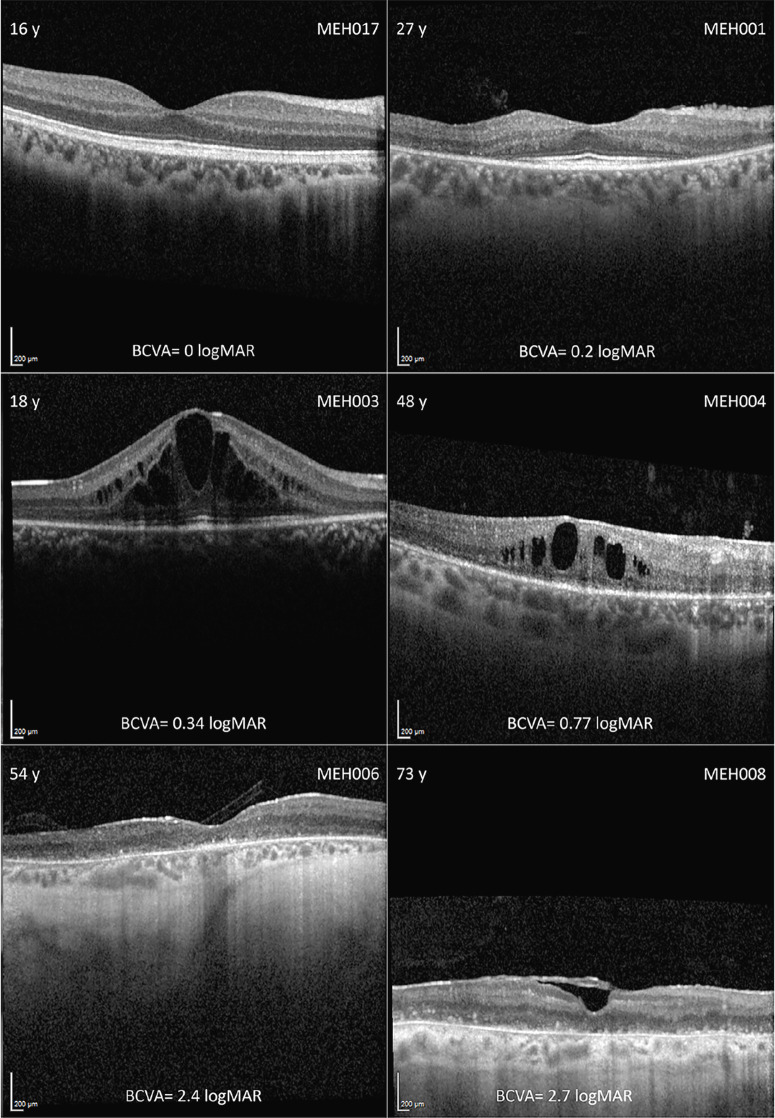
**Montage of optical coherence tomography (OCT) imaging in different disease stages.** In the *left and right top corner* are the age (years) and the subject ID, respectively, with the best corrected visual acuity (BCVA) in LogMAR below. Intraretinal cystic spaces were common across the cohort, regardless of disease stage. In late stages, there was diffuse outer retinal and retinal pigmented epithelium atrophy. Subject MEH008 also had an epiretinal membrane.

Qualitatively, 13 patients (43.3%) developed IRCS at a mean age (range; ±SD) of 27.4 years (range = 12–45, SD = ±10.6). These were mostly limited, but two patients, MEH003 and MEH008, developed significantly large IRCS (Fig. 4). Another three patients with late-stage disease had epiretinal membranes, one of whom also exhibited a lamellar hole in one eye. Eleven patients had normal architecture in the transfoveal B-scan at a mean age of 19 years (range = 3–39 years), and another 3 patients had no measurable EZW and ONL thickness at a mean age of 58.3 years (range = 49–70). MEH005, despite having sectoral-like RP, presented with considerable IRCS at his last follow-up visit that did not reduce his visual acuity (see Fig. 1A). Despite disruptions in the EZW being measured in patients as young as 6 years old (MEH010), the earliest age when the outer retina was severely atrophic (i.e. no discernible EZW or ONL), was 49 years of age; this suggests a slow progression and wide window of retinal viability from a structural perspective.

**Figure 5.**
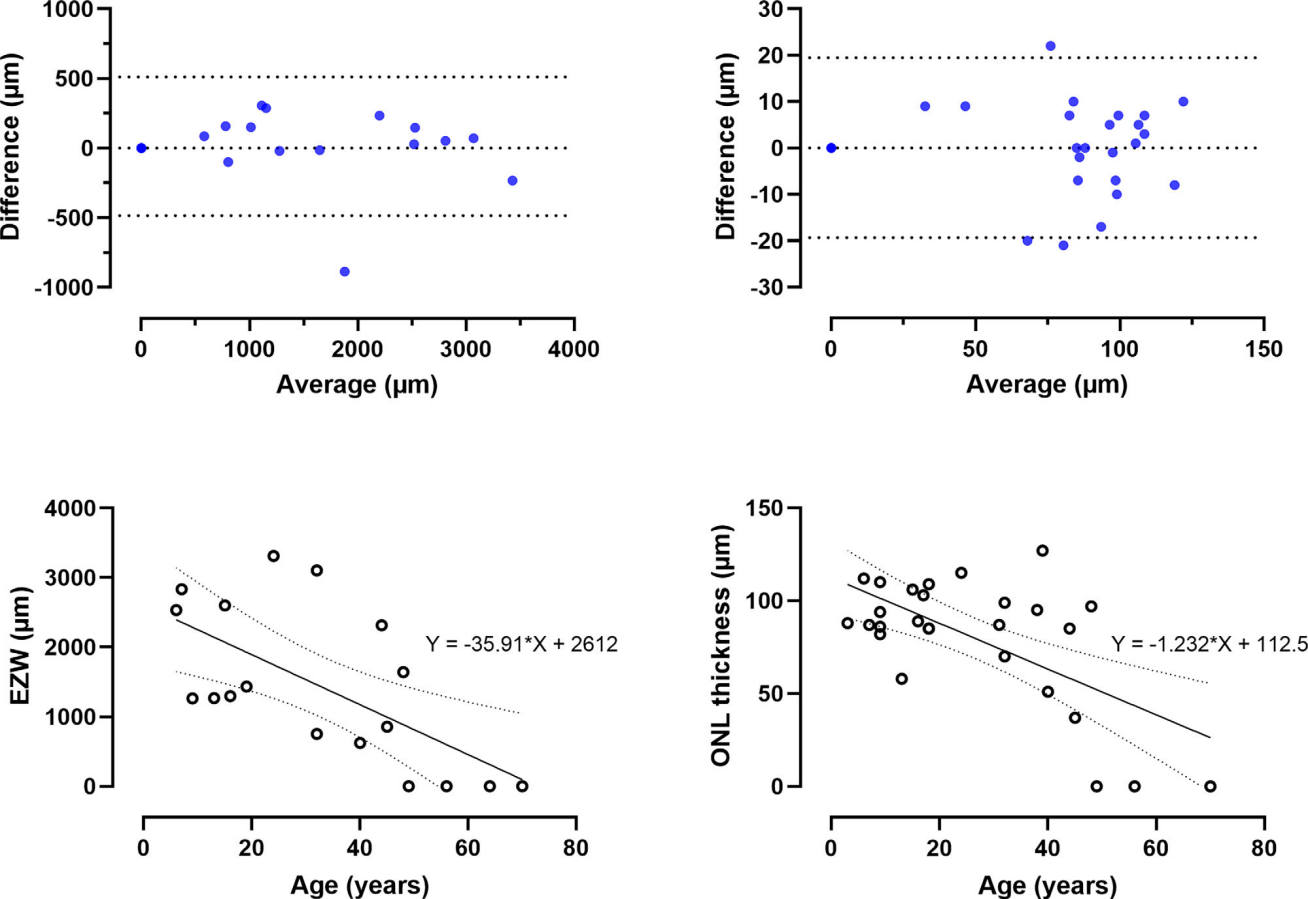
**Structural disease symmetry and change over time.** The first row shows two Bland-Altman plots of the ellipsoid zone width (EZW) and outer nuclear layer (ONL) thickness. The 95% limits of agreement are plotted as dotted lines. The bias (±SD) was 14.21 µm (±254.1) for EZW, and 0.07 µm (±9.9) for ONL thickness, revealing high interocular agreement. The second row is a simple linear regression of EZW and ONL thickness against age, revealing negative trends, both of which were statistically significant (*P* = 0.004 and 0.0004, respectively).

At baseline, the mean EZW (range; ±SD) was 1418 µm (range = 0–3314, SD = ±1094) in the right and 1404 µm (range = 0–3547, SD = ±1127) in the left eyes. There was high interocular agreement (Fig. 5); EZW had a bias (±SD; 95% confidence interval [CI]) of 14.21 µm (SD = ±254.1, 95% CI = −108.3 to 136.7) with limits of agreement of −483.9 (95% CI = −697 to −270.8) to 512.3 (95% CI = 299.2 to 725.4). The mean ONL thickness was 79.9 µm (range = 0–123, SD = ±34.7) in the right eyes and 79.5 µm (range = 0–123, SD = ±35.8) in the left eyes. ONL thickness had a bias (±SD; 95% CI) of 0.07 µm (SD = ±9.9, 95% CI = −3.9 to 4.1) with limits of agreement of −19.33 (95% CI = −26.2 to −12.4) to 19.48 (95% CI = 12.5 to 26.4). There was a very high interocular correlation in both EZW (*r* = 0.99, *P* < 0.0001) and ONL thickness (*r* = 0.89, *P* < 0.0001); hence, only the parameters in the right eyes are reported longitudinally.

In patients who had longitudinal data available, the mean EZW (range ±SD) changed from 1437 µm (range = 0–3314, SD = ±1123) to 1045 µm (range = 0–2812, SD = ±921) over a span of 7.2 years, which was significantly different (*P* < 0.0001; Wilcoxon matched-pairs signed rank test). Similarly, the mean ONL thickness reduced from 79.9 µm (range = 0–123, SD = ±34.7) to 67.6 µm (range = 0–115, SD = ±35.7), which was also significant (*P* = 0.0002; Wilcoxon matched-pairs signed rank test). This change is expected given the progressive nature of the condition, but it does suggest a slow disease progression. A simple linear regression revealed a significant inverse trend between age versus both EZW (*P* = 0.004) and ONL thickness (*P* = 0.0004; see Fig. 5).

### Electrophysiology

There was high degree of interocular ERG symmetry based on amplitudes of the ISCEV Standard DA 0.01, DA 10 ERG a- and b-waves, LA 30 Hertz (Hz) ERG and LA 3 (single flash) ERG b-waves (slope = 0.9, *r*^2^ = 0.86) and LA 30 Hz peak times (slope = 1, *N* = 7).

**Figure 6.**
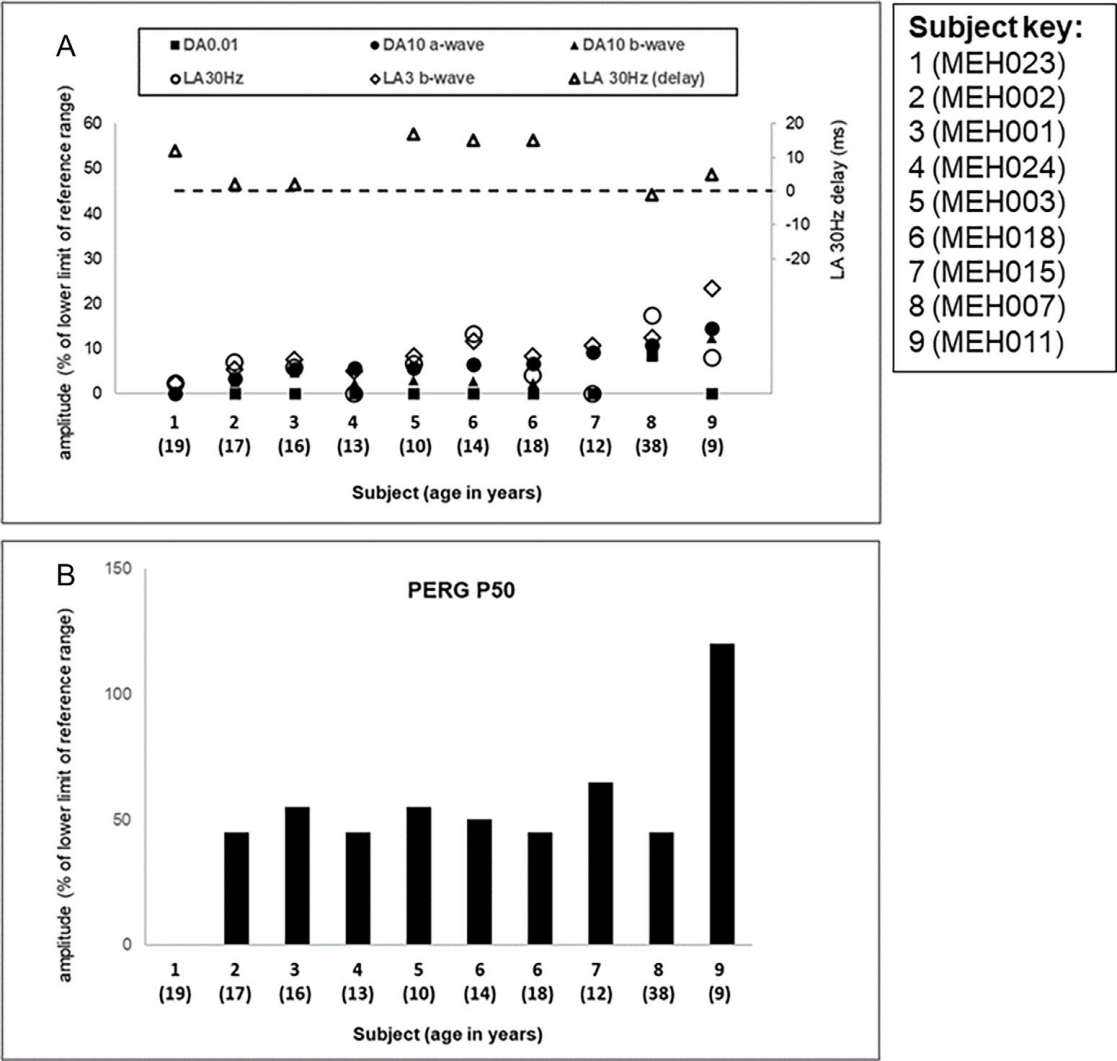
**Full-field ERG and PERG P50 findings.** Full field and pattern ERG findings summarized in nine subjects that were tested according to ISCEV standard methods. (**A**) The amplitudes of the DA0.01 ERG, DA 10 ERG a- and b-waves, LA 30 Hz ERG, and LA 3 ERG b-wave are plotted against the primary axis as a percentage of the age-matched 5th percentile of the (“normal”) reference range, with values arranged in ascending order of DA10 ERG a-wave amplitude for clarity. The LA 30 Hz peak times are plotted as a difference from the age-matched 95th percentile of the reference range (*horizontal broken line* indicates 0 ms delay) against the secondary axis. (**B**) The ISCEV Standard PERG P50 amplitudes of the same patients shown in (**A**). Note that ERG and PERG P50 data for subject 6 are shown at the age of 14 years and at 18 years.

The ISCEV Standard ERG and PERG P50 findings and patient ages at the time of testing are summarized in Figure 6. All patients had ERG evidence of a photoreceptor dystrophy with greater involvement of rod than cone systems evident in most cases (see Fig. 6A). There was delay in 5 of 7 cases with a detectable LA30Hz flicker ERG at baseline (range of delay 2 to 17 ms); 1 case showed borderline delay and 1 case had a LA30Hz ERG of normal timing (see Fig. 6A; secondary axis). Pattern ERG P50 was normal (*N* = 1), relatively preserved (*N* = 7), or undetectable (*N* = 1) at initial testing, in keeping with variable degrees of macular involvement (see Fig. 6B). Of the four children tested using skin electrodes and a shortened ERG protocol, photopic and scotopic flash ERGs were almost undetectable (*N* = 1), showed a similar degree of abnormality (*N* = 2) or were borderline (*N* = 1). PERG P50, recorded with skin electrodes (*N* = 3) or gold foils (*N* = 1), was preserved in 3 of 4 cases including the child with borderline flash ERGs (age 4 years).

Follow-up electrophysiology was available after 4.5 years in one individual with initial ERG evidence of a rod-cone dystrophy and a subnormal but detectable PERG (see Fig. 6, subject 6), tested at the ages of 14 and 18 years. DA ERGs and PERGs were stable but LA ERGs showed mild reduction in amplitudes. There was no obvious correlation between age and ERG or PERG P50 components in the group as a whole, although there was a limited age range and few adult subjects. The oldest individual tested (age 38 years) had one of the mildest ERG phenotypes (see Fig. 6; MEH007).

All nine subjects that underwent ISCEV Standard PERG had a paracentral ring of increased signal on autofluorescence imaging. The patient with an undetectable ERG had a relatively small ring and additional CMO at the time of testing.

## Discussion

This longitudinal study explores the clinical and electrophysiological phenotype, the retinal imaging and other aspects of the natural history of USH1D. To the best of our knowledge, it represents the largest cohort described cross-sectionally and longitudinally.

### Disease Onset and Course

Patients affected with USH1D have an onset of visual symptoms before 18 years of age, with more than half being symptomatic before 10 years, and 3 patients presenting with nystagmus in the first year of life. The most common visual symptoms at presentation were nyctalopia and difficulties with peripheral vision. This is in keeping with the frequently described onset of RP within the first decade of life in patients with USH type 1.^25^ Indeed, the fundus findings were compatible with typical RP, albeit three patients had an unusual RP phenotype with superior retinal sparing. Interestingly, all three harbored the c.5237G>A p.(Arg1746Gln) variant, which was previously reported in a case series of individuals with sectoral RP^21^ and may represent a hypomorphic allele. Cataracts were common and present in more than a third of our patients, frequently before the fourth decade of life. Most of those that underwent electrophysiology had evidence of moderately severe to severe rod-cone dystrophy, with PERGs consistent with relative sparing of macular function. The variants c.753G>A and c.5985C>A in *CDH23* have been associated with schizophrenia in recent studies.^26^^,^^27^ A chart review shows no evidence of the disease in any subject described herein; similarly, the aforementioned variants are not present in this cohort.

BCVA was relatively preserved throughout the disease, and although there was a significant decrease with age, only one patient below 50 years of age had vision that was worse than 1.0 LogMAR. Similarly, the rate of BCVA decrease was less than 0.02 LogMAR/year; if extrapolated, this implies that patients lose, on average, a line of vision every 5 years. There was a high interocular correlation of BCVA, suggesting the disease to be highly symmetric from a functional perspective, which is promising in the context of future therapies with novel investigational medical products, where the fellow eye might be used as a control. Further functional evaluations are necessary to confirm these findings. Interestingly, the refractive errors were usually mild with many patients with emmetropia; the most common type of refractive error being myopic astigmatism, which was seen in a third of the cohort.

### Retinal Imaging

All structural parameters herein also display significant interocular symmetry. HyperAF rings were typical on FAF imaging and seen in most patients at some point during follow-up. These are in fact present in a variety of inherited retinal dystrophies, but double rings such as the ones described herein, are rarer and to the best of our knowledge, have been reported in only a few genotypes to date, such as the retinal dystrophies associated with disease-causing variants in *ABCA4* (OMIM *601691), *NR2E3* (OMIM *604485), *EYS* (OMIM *612424), and *USH2A* (OMIM *608400).^28^^–^^31^ It is highlighted that in spite of patients having a moderately severe to severe photoreceptor or rod-cone dystrophy, most of those tested showed relative preservation of PERG P50, broadly consistent with preservation of function within the FAF rings.^32^^–^^34^ Furthermore, the hyperAF rings seemed to encroach toward the posterior pole, except when the ring was wide and extended beyond the vascular arcades. Patients with these large rings also appeared to have the mildest phenotype across the cohort, which is in keeping with the ERG findings. Qualitative analysis of the OCT revealed IRCS in more than 40% of patients, which – in the context of USH syndrome – if combined with the aforementioned FAF features and a mild refractive error mostly influenced by astigmatism, may be suggestive of *CDH23*-associated USH syndrome (USH1D).

The quantitative parameters analyzed herein suggest a wide window of retinal structural preservation and a slowly progressive natural history. Furthermore, over a third of the subjects had normal retinal architecture on the transfoveal B-scan before 20 years of age. Indeed, despite the earlier onset of symptoms, the retinal phenotype is more in keeping with that of *USH2A*, with some features overlapping. Specifically, the presence of double hyperAF rings has only been reported in a few RP-related genes.^31^ Furthermore, the slow longitudinal changes in retinal architecture resemble those previously reported in *USH2A*.^35^^–^^37^ One gap that remains relates to how the retinal sensitivity changes with disease progression, which would ultimately allow for a more specific comparison with other phenotypes, such as that of *PCDH15*-associated USH1D. This would provide an in-depth functional perspective of the condition described herein. Similarly, more extensive and standardized protocols performed longitudinally will be invaluable to better describe large cohorts of affected individuals. Similarly, further investigation is possible using imaging systems with greater resolution, such as custom-built adaptive optics scanning light ophthalmoscopy (AOSLO) devices, which would allow for a metric-derived characterization of the microscopic photoreceptor mosaic.^38^ Moreover, based on our personal experience with AOSLO, even in patients without discernible EZW and ONL, fixation may be preserved, further highlighting the role of psychophysical testing in helping establish function with fixation-derived metrics. In the 3 patients with late-stage disease (see Fig. 4), none had BCVA better than 2.3 LogMAR (i.e. counting fingers), but volume scans were still obtainable, suggesting the presence of remnant foveal cones.

One limitation of our retrospective approach is that axial length (AL) was not available for most of the cohort. This is essential for the accurate scaling of EZW because AL is known to affect the transverse scaling of OCT images.^39^

### Future Directions

Given the relatively easy acquisition and repeatability of retinal imaging, EZW and ONL thickness may indeed be broadly robust end points. The only quantifiable parameter derived from FAF imaging was the hyperAF ring, which should be interpreted with caution. This has been quantified herein with difficulty given the absence of standardization and the frequent presence of cataracts, which influenced image quality, causing difficulties to accurately delineate these rings. Pattern ERGs were used to assess macular function objectively in a cohort of subjects, although functional parameters such as retinal sensitivity measurements would be corroboratory, could provide greater spatial resolution and could be used to probe both macular cone and macular rod function. These would be useful, particularly given the possibility to establish correlations with structural end points.

From a treatment perspective, mouse models for *CDH23*-related non-syndromic hearing loss – C57BL/6J and salsa – exhibit a similar progressive hearing loss to that in humans. If these models also had a retinal phenotype similar to humans, which they do not, they would potentially be therapeutically targeted by novel treatments such as gene therapy.^40^^–^^42^ However, the main restriction for gene therapy is the size of the *CDH23* gene, which spans about 10.1 kb and is more than twice the cargo capacity of the current generation of adeno-associated viral vectors, the most commonly used vector for gene therapy. There remains, however, an enormous interest in development of novel vector technology, with some groups developing triple vectors to expand this transfer capacity.^43^

## Conclusions

To the best of our knowledge, this retrospective study is the first in-depth longitudinal study of USH1D. Despite the early onset of symptoms, our findings suggest that the natural history of macular involvement is relatively slow, and the window for intervention wide – up to 50 years of age in most cases. USH1D appears to be an arguably ideal target for novel therapies, particularly given the high interocular symmetry. There is a need for a prospective natural history study to further investigate disease progression from a functional perspective, as well as further refine clinical trial end points.
